# Human Umbilical Cord-Mesenchymal Stem Cells Survive and Migrate within the Vitreous Cavity and Ameliorate Retinal Damage in a Novel Rat Model of Chronic Glaucoma

**DOI:** 10.1155/2021/8852517

**Published:** 2021-10-25

**Authors:** Yao Wang, Jiexuan Lv, Changquan Huang, Xiaohong Li, Yongxiong Chen, Wutian Wu, Renyi Wu

**Affiliations:** ^1^Xiamen University Affiliated Eye Center, Xiamen 361100, China; ^2^Shaanxi Provincial Key Lab of Ophthalmology, Shaanxi Clinical Study Center for Ocular Disease, Shaanxi Institute of Ophthalmology, First Hospital of Xi'an, First Affiliated Hospital, Medical School, Northwest University, Xi'an 710002, China; ^3^Fujian Provincial Key Laboratory of Ophthalmology and Visual Science, Eye Institute of Xiamen University, Xiamen 361102, China; ^4^School of Biomedical Science, State Key Laboratory of Brain and Cognitive Sciences, Li Ka Shing Faculty of Medicine, The University of Hong Kong, Pokfulam, Hong Kong SAR, China

## Abstract

Glaucoma is the leading cause of irreversible blindness worldwide, and pathologically elevated intraocular pressure (IOP) is the major risk factor. Neuroprotection is one of the potential therapies for glaucomatous retinal damage. Intravitreal mesenchymal stem cell (MSC) transplantation provides a viable therapeutic option, and human umbilical cord- (hUC-) MSCs are attractive candidates for cell-based neuroprotection. Here, we investigated the ability of transplanted hUC-MSCs to survive and migrate within the vitreous cavity and their neuroprotective effects on chronic glaucomatous retina. For this, we developed a chronic ocular hypertension (COH) rat model through the intracameral injection of allogeneic Tenon's fibroblasts. Green fluorescent protein-transduced hUC-MSCs were then injected into the vitreous cavity one week after COH induction. Results showed that a moderate IOP elevation lasted for two months. Transplanted hUC-MSCs migrated toward the area of damaged retina, but did not penetrate into the retina. The hUC-MSCs survived for at least eight weeks in the vitreous cavity. Moreover, the hUC-MSCs were efficient at decreasing the loss of retinal ganglion cells; retinal damage was attenuated through the inhibition of apoptosis. In this study, we have developed a novel COH rat model and demonstrated the prolonged neuroprotective potential of intravitreal hUC-MSCs in chronic glaucoma.

## 1. Introduction

Glaucoma comprises a group of diseases causing irreversible blindness characterized by the progressive loss of retinal ganglion cells (RGCs) and their axons. Elevated intraocular pressure (IOP) is one of the most important risk factors for glaucomatous optic neuropathy. However, the common pharmacological or surgical treatments to reduce IOP do not halt progressive visual loss [1]; therefore, besides lowering IOP, alternative treatment approaches for glaucoma are needed. Current studies of cell-based neuroprotective therapies in animal models have introduced a new possibility for amelioration of retinal degeneration [2, 3].

The primary obstacle limiting stem cell therapy is the identification of an appropriate cell source. Mesenchymal stem cells (MSCs) of different origins have shown the greatest potential in the treatment of optical neuropathy [4–6]. For example, intravitreal transplantation of human umbilical cord (hUC) blood stem cells or dental pulp stem cells is neuroprotective for RGCs in rats with optic injury [6–9]. An emerging alternative is the use of hUC-MSCs, which are derived from Wharton's jelly, the particular connective tissue among the veins and arteries of the fetal cord [10–14]. hUC-MSCs have the biological features of both adult stem cells and embryonic stem (ES) cells but are closer to the original form and proliferative than are adult stem cells. hUC-MSCs are more attractive candidates than ES cells for retinal treatment and neural progenitor cells because populations of hUC-MSCs can easily be expanded and their use avoids ethical controversy [15]. In addition, these cells are readily available and can be acquired noninvasively (unlike bone marrow- (BM-) MSCs, which are harvested from the bone marrow) and can also differentiate into a variety of retinal neuron types, exhibiting extensive proliferation in vitro [16]. Altogether, these key attributes make hUC-MSCs attractive candidates for the development of cell-based neuroprotective therapies.

To study the mechanisms of glaucomatous damage, the widely used experimental approach is to induce elevated IOP in animals [17–19]. Induced ocular hypertension models include rats with injection of different substances, such as hyaluronic acid [20] or autologous ghost red blood cells [21], into the anterior chamber to block the aqueous outflow. However, the relatively short duration of such IOP elevation requires multiple injections, which hinders the application of these models for research use. Instead, we blocked the aqueous humor drainage pathway, to develop a novel chronic ocular hypertension (COH)/glaucoma model, by injection of Tenon's fibroblasts into the anterior chamber of rat eyes. Tenon's fibroblasts were taken from Tenon's capsule of allogeneic transgenic green fluorescent protein- (GFP-) expressing Sprague-Dawley rats, to trace the survival and migration of the cells in the anterior chamber of the eye. Tenon's capsule is a thin membranous socket of dense connective tissue, enveloping the eyeball and lying between the conjunctiva and the episclera.

In the present study, we developed a novel method for inducing elevated IOP and preliminarily evaluated the reliability of this rat model of chronic glaucoma. Then, hUC-MSCs marked with GFP were transplanted intravitreally, to investigate whether transplanted hUC-MSCs could survive and migrate in the vitreous cavity. We also demonstrated long-term neuroprotective efficacy of transplanted hUC-MSCs in the novel model, through the evaluation of RGC loss, retinal damage, and apoptosis in retinal cells caused by IOP elevations.

## 2. Materials and Methods

### 2.1. Animals and Experimental Groups

The study strictly adhered to the Association for Research in Vision and Ophthalmology (ARVO) Statement for the Use of Animals in Ophthalmic and Vision Research. The experimental protocols were approved by the experimental animal ethics committee of Xiamen University. Adult (8-12 weeks old) wild-type Sprague-Dawley rats were used in this study. In addition, two transgenic GFP-expressing Sprague-Dawley rats were used as donors of Tenon's fibroblasts. All rats were housed in light- and temperature-controlled conditions.

In all rats, the right eye was injected intracamerally with allogeneic Tenon's fibroblasts (suspended in PBS) and used as the experimental COH eye, and the contralateral eye was injected intracamerally with PBS and served as the control group. One week after the induction of IOP elevation, all the IOP-induced rats were randomly divided into 2 groups as follows: hUC-MSCs were washed with phosphate buffer saline (PBS) and resuspended in PBS and intravitreally transplanted to the right eyes of 24 rats (COH+hUC-MSC group). PBS was intravitreally injected to the right eyes in another 18 rats that served as the COH group. All of the rats were sacrificed after 8 weeks.

### 2.2. Isolation and Culture of GFP-Tenon's Fibroblasts

The GFP-Tenon's fibroblasts were isolated from the eyes of transgenic rat that ubiquitously expressed GFP. Briefly, the superficial layer of Tenon's capsule, attached to the sclera, in the eyes of the GFP-rat was isolated under an operating microscope, washed with Ca2+- and Mg2+-free Hank's solution (Gibco BRL, Grand Island, NY, USA) and digested by 0.2% dispase II (1.10 unit/mg, Gibco). The isolated cells were incubated in humidified 95% air/5% CO2, detached with trypsin/EDTA solution and diluted 1 : 3 upon reaching 80% confluence. Cell culture was performed in Dulbecco's modified Eagle's medium (DMEM, Gibco), supplemented with 10% FBS and 1% penicillin/streptomycin.

### 2.3. Induction of COH

GFP-labeled fibroblasts from passage 2 were used for cell injection. After general anesthesia, pupillary dilation of the right eye was achieved with 0.5% tropicamide. A suspension of Tenon's fibroblasts (20,000 cells/*μ*L) was injected into the anterior chamber using a 30-gauge needle. Care was taken to avoid any injury to tissues inside the eye.

### 2.4. IOP Measurement

IOP was measured using a TonoLab tonometer (Icare, Helsinki, Finland) in awake rats without anesthesia. Six measurements were consecutively performed according to instrument requirements, and the mean of the second through sixth readings was automatically calculated and then recorded. Such measurements were repeated five times on each eye at each time point. On each occasion, the IOP for each given rat was measured at the same time of day.

### 2.5. Toluidine Blue Staining and Transmission Electron Microscopy (TEM)

High IOP of rats was maintained for 8 weeks, and the optic nerve of the eyeball was completely removed under anesthesia. The frozen section of optic nerve was stained with 2% toluidine blue. Optic nerve morphology changes were assessed under a bright-field microscope. For TEM, following 12 weeks of elevated IOP, optic nerve tissues were sectioned and counterstained with uranyl acetate and lead citrate. Images were acquired on a JEM-2100 electron microscope (JEOL Ltd., Tokyo, Japan) operated at an accelerating voltage of 120 kV for morphology analysis.

### 2.6. Isolation and Cell Culture of hUC-MSCs

The use of the human umbilical cords for research was approved by the local ethics committee, and informed consent was obtained from all donors, following explanation of the research. The donors were pregnant women with no congenital diseases, hepatitis, syphilis, AIDS, or other infectious diseases, and the gestational ages were 37–40 weeks. The hUC-MSCs were isolated as previously described [22]. Briefly, umbilical cord fragments were washed with normal saline, and the umbilical veins, arteries, and their outer membranes were removed. Wharton's jelly was minced and treated with 0.1% type-IV collagenase (Life Technologies, Carlsbad, CA, USA). Isolated cells were collected by centrifugation and cultured in DMEM/F12 supplement with 10% FBS, 1% penicillin/streptomycin at 37°C. Cultured cells to third or fourth passage were used in the subsequent experiments.

### 2.7. Immunophenotypic Characterization of hUC-MSCs

The cells were labeled with antibodies against CD29-phycoerythrin (PE), CD44-fluorescein isothiocyanate (FITC), CD105-FITC, CD34-FITC, CD45-FITC, HLA-DR-PE, CD80-FITC, CD86-FITC, and CD31-FITC. Flow cytometry was performed using a FACS Calibur (BD Biosciences, San Jose, CA, USA), and the data were analyzed with the Cell Quest software (BD Biosciences).

### 2.8. Transduction of hUC-MSCs with GFP-Encoding Lentiviral Vectors

To label hUC-MSCs, cells were placed in 12-well plates at a density of 105 cells per well in 500 *μ*l DMEM-F12 (10% FBS) for 12 h. The GFP-gene-expressing lentiviral vectors (BioWit Technologies, Shenzhen, China) were added to the medium supplemented with 8 *μ*g/ml of polybrene. Viral particles were removed after 24 h exposure. Two days later, hUC-MSCs were assessed under an inverted fluorescent microscope (Nikon, TE2000-U, Tokyo, Japan).

### 2.9. hUC-MSC Transplantation

One week after COH induction, GFP-labeled hUC-MSCs were transplanted intravitreally into the COH eyes. After general and topical anesthesia, as described above, the hUC-MSC suspension (5 *μ*l, 20,000 cells/*μ*l) was gently injected into the vitreous cavity with a 30-gauge Hamilton needle through the pars plana of the globe. The pinhole was covered up with erythromycin ointment [23].

### 2.10. Tracking of hUC-MSCs in the Ocular Tissues

The rats were sacrificed 60 days after hUC-MSC transplantation. The eyes were enucleated and fixed in 4% paraformaldehyde for 50 minutes at room temperature and then infiltrated with 30% sucrose/PBS for 24 h at 4°C. The whole-eye tissues were embedded in optimal cutting temperature compound. The frozen specimens were sectioned (7 *μ*m thickness) along the vertical meridian of the eyeballs through the optic nerve head. Tissue preparations were processed for either hematoxylin and eosin or toluidine blue or were examined under fluorescence microscope (Leica, DM2500, Wetzlar, Germany).

### 2.11. Retrograde Labeling of RGCs with Fluorogold

The retrograde labeling of RGCs with Fluorogold was performed as previously described [24]. RGCs of both eyes were retrogradely labeled seven days before the rats were sacrificed. Rats were anesthetized and mounted in a stereotactic apparatus (RWD Life Science, Shenzhen, China), and RGC labeling was done by injecting 4% Fluorogold (Fluorochrome LLC, Denver, CO, USA; 3.0 *μ*L each) into the superior colliculus at a point 6.0 mm caudal to the bregma and 1.0 mm lateral to the midline on both sides to a depth of 5.0 mm from the surface of the skull.

### 2.12. Quantification of RGC Survival

Seven days after Fluorogold retrograde labeling, rats were sacrificed and the eyes were enucleated and fixed in 4% paraformaldehyde in PBS for 1 hour in the dark at 4°C. The retinas were dissected free, flat-mounted on a glass slide, and observed under the Leica fluorescence microscope. Manual RGC counting, by an investigator masked to the experimental procedures, was conducted in the superior, inferior, temporal, and nasal quadrants at distances of 1, 2, and 3 mm radially away from the optic nerve. Cells with intense dye staining and smaller or larger size than normal were considered to be microglia or macrophages and were excluded from the analysis [24].

### 2.13. Hematoxylin-Eosin Staining

The cornea, lens, and vitreous of the rat were carefully removed, and the posterior eyecups were embedded in optimal cutting temperature compound. The frozen specimens of rat retinas were cut into 7 *μ*m sections along the vertical meridian of the eye through the optic nerve head and stained with hematoxylin and eosin. Sections were examined under a light microscope [25].

### 2.14. Western Blot Analysis

Protein extraction and Western blots were performed as reported previously [26]. In brief, dissected retinas were homogenized in ice-cold lysis buffer (Solarbio Science & Technology, Beijing, China), and 20 *μ*g of total protein was dissolved in 12%-SDS gels, transferred to polyvinylidene fluoride membranes (Millipore, Watford, UK, USA), and incubated overnight at 4°C with primary antibodies against caspase-8 (1 : 1,000; rabbit polyclonal anti-rat; Cell Signaling Technology, Danvers, MA, USA) and *β*-actin (1 : 10,000; Sigma-Aldrich, St. Louis, MO, USA). Protein bands were detected with horseradish peroxidase-conjugated goat anti-rabbit IgG (1 : 10,000; Bio-Rad, Hercules, CA, USA) and visualized by enhanced chemiluminescence reagents and quantified with the Quantity One software (Bio-Rad).

### 2.15. Statistical Analysis

Data were expressed as mean ± standard deviation. All statistical analyses were performed using SPSS (version 10.0, SPSS Inc., Chicago Ill., USA). One-way analysis of variance (ANOVA) was performed followed by the Student–Newman–Keuls test for multiple comparisons. Statistical significance was set at *p* < 0.05.

## 3. Results

### 3.1. Development of a Rat COH Model

**Figure 1:**
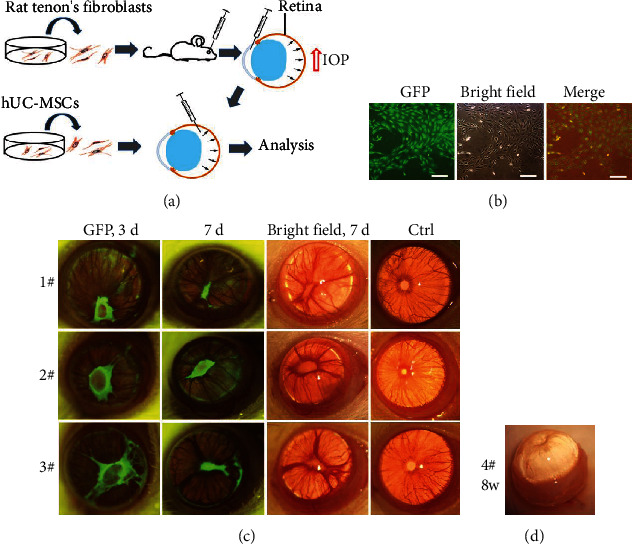
Development of rat chronic ocular hypertension (COH) models. (a) The schematic diagram illustrating the process of establishing the COH model by rat Tenon's fibroblasts and ameliorating retinal damage by hUC-MSCs. (b) Morphology of green fluorescent protein- (GFP-) Tenon's fibroblasts isolated from the eyes of a transgenic Sprague-Dawley rat that ubiquitously expressed GFP. The fibroblasts were cultured for 3 days at the second generation. Scale bar: 200 *μ*m. (c) Representative fluorescent and bright-field microscopy images (under a fluorescent microscope and an ophthalmic operating microscope, respectively) of the anterior segment of the same rat at 3 and 7 days after injection of GFP-Tenon's fibroblasts into the anterior chamber. Tenon's fibroblasts were visualized in the center within the anterior chamber by fluorescent GFP. Compared with the normal left eye (far-right column), the iris at the edge of the pupil of the treated right eye was attached to the lens and pupil atresia occurred, resulting in the iris appearing to have bulged forward, as viewed under an ophthalmic operating microscope at 7 days. (d) Representative rat eyeball at 8 weeks after injection of Tenon's fibroblasts into the anterior chamber. The panels labeled 1#, 2#, 3#, and 4# portray the anterior segment of the representative rats 1, 2, 3, and 4, respectively.

We first developed a rat COH model based on an intracameral injection of allogenic Tenon's capsule fibroblasts (Figure 1(a)). To minimize anterior-segment inflammation and track the survival and location of transplanted cells in the anterior chamber, allogeneic Tenon's fibroblasts were isolated from the eyes of transgenic GFP-expressing Sprague-Dawley rats. As shown in Figure 1(b), Tenon's fibroblasts had a spindle-like fibroblastic morphology under the inverted microscope and strongly expressed GFP fluorescence under the fluorescence microscope.

Next, the GFP-Tenon's fibroblasts were intracamerally injected. These cells were visible in the anterior chamber angle and the pupil at 3 and 7 days postinjection, under a Nikon AZ100 motorized zoom fluorescence microscope. Compared with the normal left eye, the iris at the edge of the pupil of the treated right eye was attached to the lens, resulting in pupil atresia, which may have further led to aqueous humor gathering in the posterior chamber. There was a very pronounced iris-bulge formation under ophthalmic operating microscope at 7 days (Figure 1(c)). Two weeks after the injection of Tenon's cells in the anterior chamber, GFP fluorescence gradually faded away (data not shown), indicating that the grafted cells survived for at least 7 days (Figure 1(c)). However, pupil atresia had formed through the attachment of the iris at the edge of the pupil to the lens under ophthalmic operating microscope (Figure 1(c)), and that pathological state of pronounced iris-bulge formation lasted for at least 8 weeks (Figure 1(d)).

### 3.2. Sustained Ocular Hypertension Is Induced by Tenon's Fibroblasts

To assess the reliability of the rat model, the induced elevation of IOP and the optic nerve injury were investigated. The injection of GFP-Tenon's fibroblasts into the anterior chamber (COH group) induced a prolonged and significant rise in IOP relative to the contralateral (left) eye. The increase in IOP was maintained throughout the duration of the experiment. Across animals, the mean IOP, averaged over the length of the experiment, for Tenon's fibroblast-injected eyes was 24.9 ± 6.1 mmHg, compared with 11.4 ± 0.9 mmHg for the control eyes. The peak of IOP for the injected eyes increased from 11.1 ± 1.4 mmHg to 30.9 ± 11.8 mmHg one week after the injection of Tenon's fibroblasts into the COH eyes.

**Figure 2:**
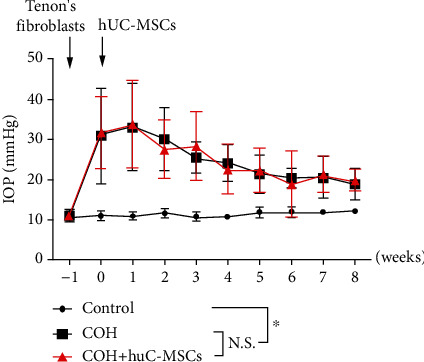
Injection of Tenon's capsule fibroblasts induced intraocular pressure (IOP) elevation. IOP was monitored for 9 weeks in the control (left) eyes and for 9 weeks following injection of Tenon's capsule fibroblasts into the anterior chamber in the right eye of each COH and COH+hUC-MSC rat. The eyes that received Tenon's fibroblasts via intracameral injection had significantly higher mean IOPs than did the control eyes (*p* < 0.05). No significant difference in the IOP profile was observed between ocular hypertensive eyes that received intravitreal hUC-MSC transplantation and the ocular hypertensive eyes that did not (*p* > 0.05). N.S.: not significant.

Additionally, 1 week after the induction of IOP elevation, hUC-MSCs were intravitreally injected into the right eye of rats in the COH+hUC-MSC group, and the IOP elevation was again assessed. Although the right eyes of rats in both the COH and COH+hUC-MSC groups had significantly (*p* < 0.05) higher mean IOPs than in the contralateral (left) eyes (control group), results showed that there was no significant difference in IOP profile, across the duration of the study, between ocular hypertensive eyes that received intravitreal hUC-MSC transplantation (COH+MSC group) and COH group that did not (Figure 2(a)). That suggests that intravitreal hUC-MSC transplantation had no effects on the IOP elevation in the Tenon's fibroblast-injected eyes.

### 3.3. Optic Nerve Injury and RGC Loss Verified the Feasibility of the COH Model

**Figure 3:**
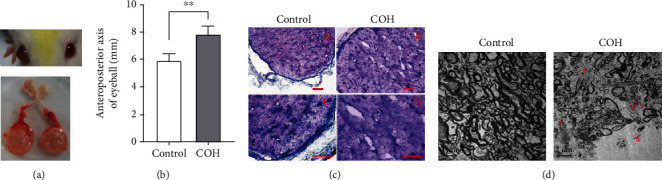
Injection of Tenon's capsule fibroblasts induced slight eye enlargement and optic nerve injury. In both the COH and COH+human umbilical cord-mesenchymal stem cell (hUC-MSC) groups, the right eyes served as treated groups and the fellow eyes served as untreated controls. (a) Slight expansion of the right eyeball, with axial elongation compared to the left eyeball, was clearly visible in macroscopic view at 8 weeks. (b) Quantitative statistics of the anteroposterior axis of eyeball in Figure 2(b). ^∗∗^*p* < 0.01. *n* = 3 for each group. (c) Toluidine blue staining images of optic nerve cross-sections from control eyes and fibroblast-injected right eyes. Intact optic nerve showed homogeneity of the staining in the control group (A, C), whereas less-stained areas indicate nerve alteration in the treated group (B, D). A, B: low magnification; C, D: high magnification. Scale bar: 100 *μ*m. COH: chronic ocular hypertension model; COH+hUC-MSC: intravitreal hUC-MSC transplantation in the chronic ocular hypertension model. (d) Transmission electron microscopy (TEM) images confirm degenerating optic nerve axons. Compared to the control group, the COH group induced by Tenon's capsule fibroblasts had extensive vacuoles (asterisk) and degenerated axons (arrows) in optic nerve. Scale bar: 2 *μ*m.

Eight weeks after injecting Tenon's fibroblasts into the anterior chamber of the rat eye, the right eye gradually expanded in macroscopic or anatomical view compared with the left eye (Figures 3(a) and 3(b)). Meanwhile, low- and high-magnification images of optic nerve cross-sections showed the homogeneity of the staining in the control group (Figure 3(c), (a) and (c)), whereas a less-stained area indicated alteration of the nerve in the treated group (Figure 3(c), (b) and (d)). Further, ultrastructural changes associated with optic nerve were examined using transmission electron microscopy (TEM). Severe demyelination of optic nerve axons was observed in 12-week fibroblast-injected eyes compared to the control group. COH-induced axonal lesions included hypomyelinated axons and many residual empty vacuoles (Figure 3(d)).

**Figure 4:**
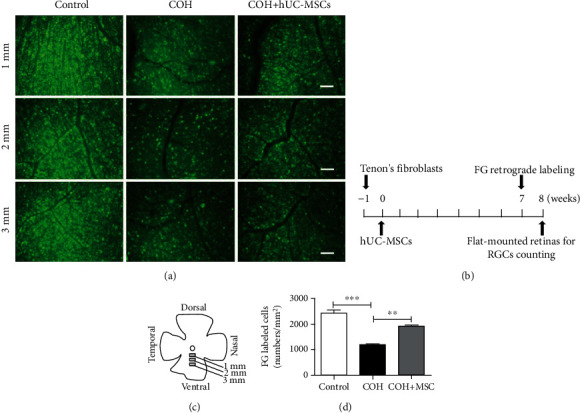
Neuroprotective effects of hUC-MSCs on RGCs in the COH model. (a) Representative fluorescence micrographs of flat-mounted retinas depicting Fluorogold-labeled RGCs in normal eyes and in glaucomatous eyes in the absence or presence of hUC-MSCs. The panels labeled as 1, 2, and 3 mm correspond to the areas marked on the whole-mounted retinas in (c). Scale bar: 50 *μ*m. (b) Schematic diagram showing the experimental protocol for retrogradely labeling RGCs with Fluorogold. (c) Schematic diagram showing the area in which Fluorogold-labeled RGCs were observed and counted on the flattened retinas. The circles shown at 1, 2, and 3 mm represent areas located 1, 2, and 3 mm radially distant from the optic disk. (d) Quantitative statistics of the numbers of labeled RGCs in the different experimental groups. RGCs were counted in 12 microscopic fields of identical size (1.19 mm^2^ areas) for each retina. ^∗∗∗^*p* < 0.001 and ^∗∗^*p* < 0.01. *n* = 3 for each group. FG: Fluorogold.

**Figure 5:**
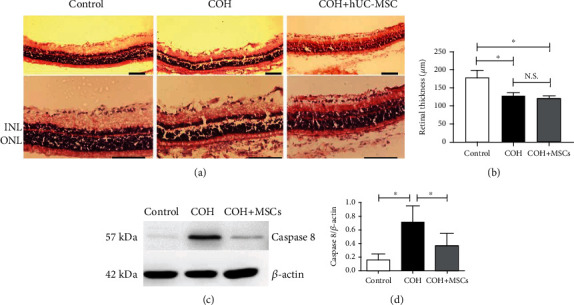
hUC-MSCs alleviate retinal damage and inhibit apoptosis of retinal cells in the COH model. (a) Hematoxylin and eosin staining of retinal histological cross-sections and (b) column chart showing retinal thickness in the normal, COH, and COH+hUC-MSC groups at 8 weeks. Scale bar: 100 *μ*m. (c) The expression of caspase-8 in retinas was determined with Western blots. (d) Quantitative analysis of the expression of caspase-8 in retinas in the different experimental groups. Duplicate detections were performed, and three eyes were included in each group. ^∗^*p* < 0.05. N.S.: not significant. GCL: ganglion cell layer; IPL: inner plexiform layer; INL: inner nuclear layer; ONL: outer nuclear layer.

Moreover, subsequent experimental results, including the loss of RGCs marked by retrograde Fluorogold labeling, the thinning of the retinal layer, and the appearance of retinal cell apoptosis caused by anesthesia (see Figures 4 and Figure 5, below), further proved the feasibility of the model.

### 3.4. Characterization of Isolated hUC-MSCs

**Figure 6:**
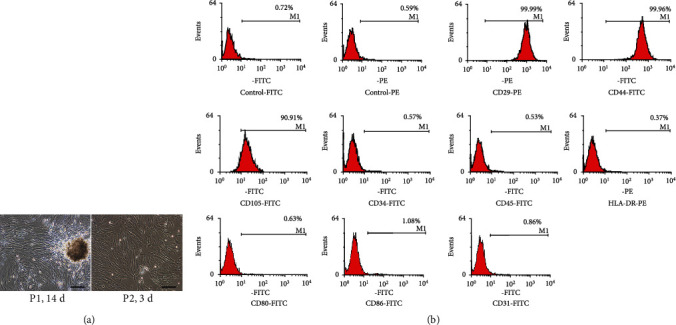
Characterization of isolated hUC-MSCs. (a) Morphology of hUC-MSCs were recorded at passage 1 (P1) and passage 2 (P2) under a microscope. Scale bars: 200 *μ*m. (b) Immunophenotypic characterization of hUC-MSCs by flow cytometry. The cells were stained with antibodies against CD29-PE, CD44-FITC, CD105-FITC, CD34-FITC, CD45-FITC, HLA-DR-PE, CD80-FITC, CD86-FITC, and CD31-FITC. The hUC-MSCs expressed CD29, CD44, and CD105 but lacked expression of CD34, CD45, HLA-DR, CD80, CD86, and CD31. CD: cluster of differentiation; HLA-DR: human leukocyte antigen-DR isotype; FITC: fluorescein isothiocyanate; PE: phycoerythrin.

The hUC-MSCs exhibited atypical spindle-shaped fibroblast morphology at passage 1 (P1). At passage 2 (P2), the cells displayed a parallel or whirlpool-like arrangement when they reached 80% confluence (Figure 6(a)). Flow cytometry (Figure 6(b)) showed strong positive staining of specific stem cell surface markers CD29, CD44, and CD105, but negative staining of human leukocyte antigen HLA-DR, hematopoietic markers CD34 and CD45, endotheliocytic marker CD31, and costimulatory molecules CD80 and CD86. These results showed that the morphology and cell-surface markers of the cells cultured by the method described above were consistent with the characteristics of stem cells.

### 3.5. Labeling of hUC-MSCs with High Efficiency and Low Toxicity

**Figure 7:**
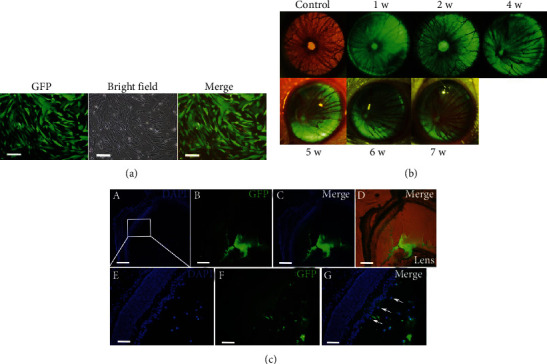
Lentivirus GFP-labeled hUC-MSC survival and localization after intravitreal transplantation. (a) Morphology of the hUC-MSCs transfected with lentivirus GFP showed the high rate of transduction, viewed under an inverted fluorescence/bright-field microscope. Scale bar: 200 *μ*m. (b) Representative fluorescent imaging under a Nikon AZ100 motorized zoom fluorescence microscope showed that GFP-labeled hUC-MSCs survived in the posterior segment for at least 8 weeks posttransplantation. (c) Fluorescence microscopy revealed that most GFP-hUC-MSCs had adhered to the posterior lens capsule, and a portion of the cells had migrated to the host retina, in clusters, within the vitreous cavity 1-week posttransplantation (A–D). Some of the GFP-hUC-MSCs had migrated to the retina and were localized in the inner limiting membrane of the retina (E–G), as indicated by the arrows (G). Low magnification images are shown in panels (A–D), scale bar: 500 *μ*m; high magnification of the area of the white quadrilateral in (A) is shown in (E–G), scale bar: 100 *μ*m.

GFP labeling, a simple and efficient technique, was applied to track the hUC-MSCs in the vitreous cavity. The efficiency of lentiviral transduction for labeling hUC-MSCs was about 85%, as observed under an inverted fluorescent microscope. The morphologic and biologic characterizations of the infected hUC-MSCs did not change, and the cells were in good condition after transduction (Figure 7(a)).

### 3.6. Survival and Migration of hUC-MSCs within the Vitreous Cavity

To perform an in vivo assessment of the long-term survival of hUC-MSCs in the vitreous chamber after cell grafting, hUC-MSCs were labeled with the GFP-encoding lentiviral vector and monitored using a Nikon AZ100 motorized zoom microscope (Figure 7(a)). Fluorescence analysis confirmed that intravitreally transplanted hUC-MSCs survived well for at least 8 weeks (Figure 7(b)). Frozen sections of the eyeball showed that hUC-MSCs deposited in clusters on the posterior surface of the lens and migrated toward the retina within the vitreous cavity by 7 days after transplantation. A small fraction of cells was seen on the inner limiting membrane of the retina (Figure 7(c), arrows). However, there was no histological evidence that GFP-hUC-MSCs integrated into the retinal layer. These findings suggested that hUC-MSCs can migrate to the site of retinal injury and survive for a long time in the vitreous cavity.

### 3.7. hUC-MSCs Alleviate the Loss of RGCs Caused by High IOP in the COH Model

To investigate the effects of hUC-MSCs on RGC survival following IOP elevation, the fluorescent tracer Fluorogold was used for retrograde labeling. Schematic diagram shows the area in which Fluorogold-labeled RGCs were observed and counted on the flattened retinas. The circles shown at 1, 2, and 3 mm represent areas located 1, 2, and 3 mm radially distant from the optic disk (Figures 4(b) and 4(c)). Quantitative analyses indicated that the labeled RGC density in control retinas was 2454 ± 190 cells/mm^2^, whereas 8 weeks after induction of high IOP, RGC numbers were reduced to 1187 ± 44 cells/mm^2^. In eyes that received hUC-MSC transplantation, the RGC density was 1926 ± 81 cells/mm^2^, significantly higher than those without hUC-MSC transplantation (*p* < 0.05, Figures 4(a) and 4(d)).

### 3.8. hUC-MSCs Alleviate the Retinal Damage Caused by High IOP

In histological comparisons with the normal control group, several changes were observed in the COH group, as follows. There was thinning of the total retina, the ganglion cell layer (GCL), the inner plexiform layer (IPL), and the outer nuclear layer (ONL), and the number of RGCs had decreased by approximately 40%. In addition, the structures of the IPL and ONL were damaged by intraocular hypertension at 8 weeks in the COH model group. In contrast, the number of RGCs was significantly higher in the COH+hUC-MSC group than in the COH group. However, no differences were observed in the IPL and the ONL between the COH+hUC-MSC and COH groups, and the thicknesses of the IPL and ONL were consistent with that in the COH model group (Figures 5(a) and 5(b)). The GCL was damaged, and the number of RGC was significantly reduced in the COH group. In the COH+HUC-MSC group, retinal damage in the GCL was reduced, and the number of RGC was higher than that in the COH group. These results suggest that hUC-MSCs had protective effects on RGCs and against GCL injury, but had no effects on IPL and ONL injury caused by high IOP.

### 3.9. hUC-MSCs Inhibit Apoptosis of Retinal Cells in the COH Model

Next, to investigate whether hUC-MSCs could improve the survival of RGCs and alleviate the damage to the retinal cells, by inhibiting apoptosis signaling in the COH model, and to explore the involved mechanisms, the level of active caspase-8 in retinas was analyzed. As shown in Figure 5(c), intravitreous injection of hUC-MSCs obviously decreased the level of active caspase-8 in the COH model relative to the untreated groups (Figure 5(d)).

## 4. Discussion

In the present study, to test the neuroprotective effect of hUC-MSC transplantation, we first developed a rat model of COH/glaucoma by injecting cultured Tenon's fibroblasts into the anterior chamber of the eye. The results revealed that hUC-MSCs survived for at least 8 weeks that they had the ability to migrate preferentially toward the damaged site of the host retina in the vitreous cavity, and that they significantly reduced retinal damage and RGC loss. These findings suggest that there is a neuroprotective effect of hUC-MSCs in an animal model of glaucoma.

Animal models are among the most useful tools for understanding disease pathology and for developing interventions against disease. To date, many rodent models have been reported to mimic the natural cause of glaucoma. In our study, rats were chosen as the animal model, in part because rats have similarities with humans in the anatomical features of the ocular anterior segment, aqueous fluid circulation, and optic nerve damage with elevated IOP [27]. The results revealed that the stable and moderate IOP elevation lasted for at least 8 weeks, with an average IOP of 24.9 ± 6.1 mmHg. Morphological assessment of the eyes with COH confirmed characteristic glaucomatous changes including the loss of RGCs. In combination with subsequent findings regarding the loss of retinal RGCs, the thinning of the retina, and the appearance of retinal cell apoptosis caused by elevated IOP, our results suggest that the experimental rat model meets the requirement for a chronic glaucoma model.

Our findings point to a plausible explanation for the elevated IOP in this novel model. The initial IOP elevation in this model may be the consequence of the aqueous humor drainage obstruction, due to the accumulation of fibroblasts in the anterior segment of the eye. One week after intracameral injection of fibroblasts, pupil atresia occurred, which might be the primary cause of the elevated IOP that lasted for approximately 2 months. In contrast with other methods to maintain elevated IOP [20, 21, 28, 29], this model may have some advantages, including being simple to carry out, only requiring a single operation, and meeting the standards of low cost and moderately high IOP with a long duration and stability. Nevertheless, due to pupil occlusion, the visual axis was obstructed during in vivo observation of the retina or optic disc with a fundoscope or fundus-imaging instrument. That occlusion is a major drawback of this animal model.

With the COH model established, we transplanted hUC-MSCs into the eyes and evaluated the changes in retinal morphology, RGC loss, and caspase-8 expression. Fluorescence from GFP-labeled hUC-MSCs could be detected inside the eye even 8 weeks after cell transplantation, indicating the survival of hUC-MSCs in the vitreous cavity. This result was similar to other reports showing that MSCs could survive for 5 weeks in the vitreous cavity in a glaucoma model [6], and that neurotrophin-4-engineered MSCs were able to survive for at least 3 months after intravitreal injection in a mouse model of acute retinal injury [30]. Because intravitreal injection is an invasive procedure and may cause complications, it is most advantageous to perform only a single injection with sustained neuroprotection.

However, although 2-month persistence was observed for hUC-MSCs in the vitreous, it is noteworthy that the Tenon's fibroblasts injected in the anterior ocular chamber persisted for less than 2 weeks, which is in line with other findings that transplanted MSCs persisted in the anterior chamber for only a few days [31]. This may be attributed to different cells (Tenon's fibroblasts versus hUC-MSCs) that survived in different microenvironments (the anterior ocular chamber versus the vitreous cavity), leading to different cell fates at different time points.

Another finding in this study is the fluorescence evidence that some of the cells migrated to the retina as clusters 1-week posttransplantation, indicating that the injected hUC-MSCs had the ability to migrate preferentially toward the damaged site of the host retina, that is, in addition to the finding that the hUC-MSCs can survive a sufficiently long time within the vitreous cavity to generate a physiological effect by secreting neurotrophic factors. Together, these results imply that hUC-MSC transplantation through the vitreous cavity is feasible. This may have important implications for the development of cell-based neuroprotective therapies.

Although our results demonstrated the migration of GFP-hUC-MSCs to the surface of the retina in the COH eyes, integration of hUC-MSCs with the retinal tissue could not be confirmed. In contrast to our results, a previous study reported that a limited number of transplanted adipose tissue and bone marrow-derived MSCs [23] did integrate into the GCL and INL of the retina. Nevertheless, our results are consistent with the concept that intravitreally transplanted cells do not, in general, migrate into the neural retina [32–34]. Penetration through the inner limiting membrane could be the major obstacle for stem cell integration into the host retina. Future studies need to be carried out to facilitate the penetration and integration of hUC-MSCs into the retina, and the consequent effect of this integration needs to be further investigated.

Intravitreally transplanted MSCs have been shown to provide a neuroprotective effect in different animal models of glaucoma. Adult bone marrow MSC transplantation increased RGC survival by about 25% at 4 weeks in a rat model of ischemia-reperfusion injury [33]. In a COH rat model induced by episcleral vein ligation, transplanting bone marrow MSCs increased RGC survival by about 15% 1 month later [32]. In the present study, for the first time, we confirmed the long-term neuroprotective potential of hUC-MSC graft in a rat model of glaucoma with a prolonged (2 months) elevation in IOP. Our results showed that the loss of Fluorogold-labeled RGCs was partially inhibited 8 weeks after hUC-MSC transplantation, and that the histological damage to the retina was less than that in the COH eyes. These findings strongly suggest that hUC-MSC transplantation into the vitreous cavity might be a therapeutic approach for the treatment of chronic glaucoma.

Accumulating evidence has shown that caspase signaling-dependent apoptosis plays a key role in the development and progression of glaucoma [35, 36]. The activation of caspase-8 is considered to be one of the initiating steps, and activation of caspase-8 and caspase-3 is representative sequential event in the apoptosis cascade of RGCs [37]. Our study showed that hUC-MSC transplantation inhibited the caspase-8 protein expression. Combined with the experimental results, including retrograde labeling of RGC with the fluorescent tracer Fluorogold, Western blot analysis of the retina, and hematoxylin and eosin staining of the cross-section of the retina, it indicated that the RGC of the retina underwent apoptosis in the COH model. hUC-MSCs could reduce the apoptosis and loss of RGC in the retina of this model, suggesting that apoptosis inhibition might be involved in the mechanism by which hUC-MSC transplantation provides retinal neuroprotection in COH eyes.

Nevertheless, other mechanisms likely also contributed to the neuroprotective effect of hUC-MSCs. For example, the cytokines and neurotrophic factors secreted from MSCs, such as brain neurotrophic factor, ciliary neurotrophic factor, and basic fibroblast growth factor, have been shown to provide neuroprotective effects in experimental models of glaucoma [12, 32]. The profile of cytokine and neurotrophic factor production after hUC-MSC injection needs to be explored further.

The eye is considered a site with relative immune privilege [38], and hUC-MSCs are reported to possess immune-privilege properties [39]. Hence, although the hUC-MSCs we used in this study were of human origin, immunological rejection of the transplantation was only a minor consideration and immune suppressants were consequently not used in our study. In fact, our results showed that no obvious adverse reactions occurred during the 2-month graft periods, suggesting the safety of heteroplastic transplantation of hUC-MSCs in the treatment of glaucoma.

## 5. Conclusions

Our study showed that a single injection of allogeneic cultured Tenon's fibroblasts into the anterior chamber of the eye resulted in a moderate and sustained elevation of IOP, damaged the optic nerve, and caused characteristic glaucomatous RGC loss in the rat retina. This seems consistent with some features of chronic glaucoma and may be useful for exploring the pathogenesis of chronic glaucoma. Intravitreally injected hUC-MSCs survived for a prolonged period and migrated toward the injured site of the retina in the vitreous cavity, without penetrating the inner limiting membrane into the retina. The hUC-MSC injections improved RGC survival, as well as ameliorating retinal damage, by inhibiting apoptosis caused by high IOP in this COH rat model. Further investigations are needed to enhance the ability of hUC-MSCs to integrate into the damaged retina and to understand the mechanisms underlying the neuroprotective effect of hUC-MSC transplantation.

## Data Availability

The data used to support the findings of this study are available from the corresponding author upon request.
